# Truth to Action: Lived Experiences of Indigenous Healthcare Professionals Redressing Indigenous-Specific Racism

**DOI:** 10.1177/08445621241282784

**Published:** 2024-10-04

**Authors:** Mona Lisa Bourque Bearskin, Meste'si Llucmetkwe Colleen Seymour, Rose Melnyk, Melba D’Souza, Judy Sturm, Tracy Mooney, Nikki Rose Hunter-Porter, Audrey Elaine Ward, Blythe Bell

**Affiliations:** 1 Beaver Lake Cree Nation, BC IHNR Chair, Associate Professor, School of Nursing, 8205 University of Victoria, Kamloops, BC, Canada; 2 Tk'emlúps te Secwépemc (Secwepemc Nation), Knowledge Holder BC IHNR, Kamloops, BC, Canada; 3 St’uxwtews (Secwépemc Nation), Executive Director, Indigenous Health & Cultural Safety, 60299 BC Mental Health & Substance Use Services, Kamloops, BC, Canada; 4 Associate Professor, School of Nursing, 33527 Thompson Rivers University, British Columbia, Kamloops, BC, Canada; 5 Pellt'iq't First Nation, Senior Practice Leader, Chee Mamuk, 113269 BC Centre for Disease Control, Kamloops, BC, Canada; 6 Métis Nation BC Citizen and guest on Syilx Lands, Regional Human Resources Director, BC First Nations Justice Council, Kamloops, BC, Canada; 7 St’uxwstews (Secwépemc Nation), Research Associate, BC IHNR, Clinical Nurse Specialist, Kamloops, BC, Canada; 8 Upper Nicola Band (Syilx Nation), Research Participant and Advisory Member, Practice Lead, MHSU NetWork, Victoria, BC, Canada; 9 Research Associate, BC IHNR Chair, 8205 University of Victoria, Victoria, BC, Canada

**Keywords:** Indigenous health, Indigenous healthcare professionals, Indigenous wellness knowledge, ‌distinction base healthcare, decolonization, cultural safety, cultural humility, Indigenous health systems transformation

## Abstract

**Study Background:**

The experience of discrimination through stereotyping, profiling, and bias-informed care not only leads to poor access to healthcare services, but low retention rates of Indigenous health professionals (IHP). As health systems transformation evolves, a significant gap remains in supporting IHP to safely address racism, to be supported culturally to bring their authentic selves and voices to work, and to attend to one's own intellectual, physical, relational, cultural and spiritual wellness within a westernized model of care.

**Purpose:**

The aim of the study was to investigate the experiences of IHP working in mainstream healthcare in order to understand how their work environment impacts the delivery of cultural safe practices. What is reported in this manuscript, as an exercise in truth-telling, is findings about lived experiences of IHP working in one mainstream provincial healthcare region, and not the whole context and outcomes of the study.

**Methods:**

Using Indigenous research methodologies, we embodied our Indigeneity into every facet of the research process. We facilitated three talking circles with participants grounded in a distinct cultural and ceremonial context following Secwepemc protocols.

**Results:**

The collective voices of IHP revealed the following common experiences: confronting genocide; addressing Indigenous-specific racism; uprooting toxicity and inequities; and upholding Indigenous human rights while enhancing accountability of systems transformation.

**Conclusions:**

The experience of IHP working in health systems goes beyond mere individual employment obligations, its often about a families and communities advocacy for Indigenous rights, culturally safe working environments and access to dignified and respectful healthcare service. This study highlights the need for IHP to be actively involved in health system transformation to ensure the redesigning and restructuring of healthcare service delivery by and for Indigenous Peoples remains centered on Indigenous health and human rights.

“*Despite the lingering effects of racism fatigue, our Peoples are strong and hold distinct traditional systems of care rooted in the land. Roots that make the baskets that hold our stories, carry our salmon, berries, and medicines to sustain our ancestral well-being, and right to health”. Colleen Seymour, Tk'emlu'ps te Secwe'pemc Member 2022*

We begin this article by acknowledging the ancestral lands and experiences of Indigenous families whose histories, knowledge, languages, cultural and spiritual practices inform our individual and collective consciousness. Four of the authors were born and raised in the homelands of Secwepemcúl'ecw. The remaining authors self-locate as a member of BC Métis Nation, a Cree Nurse from Treaty 6, two settler scholars living as uninvited guests within the same territory where the study took place. and a third settler scholar living on the Lekwungen, Coast Salish territory. All authors express their commitment to upholding Indigenous rights and advancing social justice while working in partnership. It is because of this commitment and connection we all honor and express our deepest gratitude for the relationships established and the research responsibility and accountability that we now hold to the First Nations, and Métis Peoples involved in this study.

## Introduction

Current healthcare systems and structures are colonial, hierarchical, and built on Eurocentric patriarchal ideologies where racial discrimination is maintained (National Collaborating Centre for Indigenous Health, 2014; Seymour et al., 2023). Western health philosophies remain dominant over Indigenous wellness models, creating barriers for Indigenous people seeking health services that are respectful of their ways of being, knowing, and doing (Greenwood et al., 2018; Reading & Wien, 2009). These colonial foundations continue to exacerbate inequities that re-harm and re-traumatize Indigenous Peoples seeking and delivering those services, and over time has caused distrust and burnout in the healthcare system resulting in inappropriate, unresponsive, and culturally unsafe care (Allan & Smylie, 2015; Browne et al., 2005; Ray et al., 2022). The broader British Columbia (BC) healthcare system has yet to fully recognize and uphold Indigenous rights under international, federal and provincial law (Jongbloed et al., 2023). Hundreds of recommendations voiced by Indigenous Peoples, including those in the National Inquiry into Missing and Murdered Indigenous Women and Girls (MMIWG) report (2019) and the Truth and Reconciliation Commission's (TRC) Calls to Action (2015b), outline steps required to meet the holistic wellness needs of Indigenous Peoples. Since the release of the TRC nine years ago, 81 calls remain un-actioned, which protects the status quo in these institutions and systems (Jewell & Mosby, 2023).

The TRC's (2015b) 23rd call to action states: “We call upon all levels of government to … increase the number of [Indigenous] professionals working in the health-care field” (p.3). The First Nations Health Authority makes a similar recommendation in their policy statement on cultural safety and humility stating the need to “support First Nations health care professionals to work in First Nations communities; develop initiatives to recruit and retain First Nations health leaders, health care professionals and other employees; and encourage First Nations students to become health professionals” (FNHA, 2016, p. 17). Of the Indigenous health professional (IHP) population in Canada, 74.5% are registered nurses, and yet only 3% of the Canadian registered nursing workforce are Indigenous (University of Saskatchewan, 2018). Indigenous healthcare workers are similarly disproportionately low in health systems of Australia, New Zealand, and the US (Durey et al., 2012; Lambert et al., 2014). A lack of health leadership in implementing a national Indigenous workforce strategy (Richmond & Cook, 2016) is reflected in these disproportionate numbers of Indigenous nurses and health professionals (Canadian Nurses Association, 2014). Ashworth (2018) and Macaulay (2009) both reflect that IHP representation remains very low despite current recruitment and retention efforts in their contexts, so questions of workplace conditions ought to arise as a line of inquiry. IHP working within these colonial systems share distinctive lived and living experiences that may include experiences of Indigenous-specific stereotyping, discrimination, and racism that have profound impacts on both their personal and professional lives (Dion Stout et al., 2021).

The purpose of this study was to address this gap in scholarship and knowledge by exploring the experiences of IHP^1^ working in mainstream healthcare roles in one health region in BC. Our interdisciplinary approach to redressing Indigenous-specific racism became about our collaborating to co-create an IHP supportive network with and for each other within our workplaces, as we engaged in this research. Together we co-created mutual understandings of what it meant to be a First Nations or Métis health professional using a local process grounded in Secwépemc protocols. Co-researchers were committed to working towards combining their own Indigenous knowledge systems and health expertise from a place of experience to better meet the health needs of First Nations, Métis, and Inuit Peoples they serve. To honor these collective experiences, it was imperative to create dedicated space in the research and in this manuscript to talk about the truths shared as a means of recognizing the colonial violence. More importantly our team saw it as an act of self-determination and sovereignty over political and social health transformation. In concluding our conversations this statement by one of the co-researchers was echoed by all IHP:

[what we need]…is research and policy done around retention because we work hard to get Indigenous staff into the door… but then what are we doing to help them feel safe and to keep them in the organization so they don't want to leave because they’re not supported in their own cultural values and beliefs?

## In the Literature

A lack of IHP has consequences for care. A systematic review by Gibson et al. (2015) determined the presence of Indigenous health workers promotes culturally safe care for Indigenous patients. Lambert et al. (2014) suggest Indigenous healthcare professionals combat cultural barriers currently present within the healthcare systems of Canada, Australia and New Zealand. Research by Anderson and Lavallee (2007), Ashworth (2018), Macaulay (2009), Sanzone et al. (2019), and Smith et al. (2017) all conclude that IHP are required to foster engagement, build trust, and ultimately improve the services the health systems provide to Indigenous Peoples. Further, studies report that IHP better support Indigenous patients, provide more holistic care, make it safer for patients to ask questions, improve ease of access to healthcare services, and act as interagency advocates for patients’ cultural needs (Bond et al., 2019; Bourque Bearskin et al., 2016; Browne et al., 2016; Durey et al., 2012; Gibson et al., 2015; Hartz et al., 2019; McCalman et al., 2019; Stuart & Gorman, 2015)

Van Bewer et al. (2020) highlight the critical role of Indigenous perspectives in nursing, emphasizing their alignment with the profession's core ethical values of relationality and holism. Their study suggests potential benefits for both students and educators, fostering representational and transformational growth. Vukic et al. (2012b) address the underrepresentation of Indigenous Peoples in health professions by exploring Indigenous identity and the nursing work-life quality data from 22 Aboriginal nurses in Atlantic Canada revealing six major themes: cultural context of work-life; becoming a nurse; navigating nursing; race, racism and nursing; socio-political context of Aboriginal nursing; and way forward. Monchalin et al. (2020) state that colonial policies and identity debates have led to significant gaps in culturally safe health and social services for Métis Peoples in Canada. Their research explores 11 urban Métis women's identity and their experiences with these services in Toronto through connections to community, intergenerational survival strategies, a learning journey, and a connection to land. Vukic et al. (2012a) show that nurse researchers trained in Euro-Western traditions are recognizing the significance of Indigenous knowledge systems and research methodologies and they highlight “two-eyed seeing,” which integrates Indigenous and Western perspectives in research, fostering ethical spaces for co-creating knowledge. Such an approach aligns with local traditions, blending Indigenous and Western worldviews.

Bond et al. (2019) reviewed more than 40 years of literature on Indigenous Australian healthcare workforce and found that most of the literature is limited to questions of “supply” despite the presence of additional issues such as the gaps in recognition of Indigenous knowledges, in organizational and governance structure, and in “self-awareness by health professionals of their whiteness” (p. 389). Some of this literature is concerned with retaining and supporting nursing students through the “reality shock” that new Indigenous nursing graduates face in the workplace. A resounding theme of Bond et al.'s literature review is racism; attributed as institutional racism, unconscious biases and cultural insensitivities perpetuated by systems. Bond et al. (2019) found that experiences of “blatant racism, degradation, training delays, bullying, harassment and racial vilification” (p. 392) are common amongst Indigenous healthcare professionals. The need to “better support” Indigenous workers, a commonly offered solution within the literature, is critiqued by Bond et al. because it does not acknowledge how institutional conditions contribute to Indigenous workers’ need for support. To understand the experiences of Indigenous healthcare professionals, one must consider that power operates the same way for IHP as it does for Indigenous patients. Therefore, initiatives to empower IHP must be conducted at an organizational/institutional level. Currently there is a significant gap in supporting IHP to safely address Indigenous-specific racism, to incorporate cultural practice that allow Indigenous Peoples to bring their authentic selves and voice to work, to debrief within a safe network, and to attend to their wellness in mind, body, and spirit within the westernized model of care as protective factors and restorative strategies (Askew et al., 2020; Martin-Hill, 2009).

## Setting the Context

This study was located within Interior Health Authority (IH), one of BC's five geographic health authorities, with employees of the health authority. Located in the southern interior region of BC, IH serves more than 840,000 people and covers more than 215,000 square kilometers of the ancestral, unceded, and traditional territories of seven First Nations: Dãkelh Dené; St’át’imc; Syilx; Tŝilhqot’in; Ktunaxa; Secwépemc; and Nlaka’pamux (IH, 2020). With 54 diverse and distinct First Nations communities and 15 Métis Chosen Chartered Communities, the Indigenous population constitutes approximately nine percent (63,855) of the region's population.

IH recently established an Aboriginal^2^ Employee Experience Department within the Human Resources framework, aiming to create a robust diversity and inclusion strategy platform. This initiative is dedicated to ensuring that Aboriginal employees in the organization encounter traditional and culturally safe work practices and experiences. IH is committed to achieving a 10 percent representation of the Aboriginal workforce by the year 2025, with the current self-identification standing at 6.7% (IH, 2023). In response to influential reports and calls to action, such as the In Plain Sight report (BC Ministry of Health, 2020), the TRC Calls to Action (2015b), MMIWG Calls to Justice (MMIWG, 2019), and the Declaration on the Rights of Indigenous Peoples Act and its Action Plan (Government of British Columbia, 2019, 2022), IH is actively developing an Aboriginal Employee Experience Strategy. This strategic initiative builds upon the existing IH Aboriginal Health Human Resources Plan with the ongoing enhancement focused on programs and initiatives geared towards recruiting and retaining Aboriginal employees, establishing an anti-racist, culturally safe, and inclusive work environment, and supporting Aboriginal leadership development. Aligned with IH's Strategic Priorities to support Aboriginal health and wellness and foster an improved and inclusive culture (IH, 2023), these key directives are vital for ensuring the overall health and well-being of IH's Aboriginal workforce, while simultaneously improving recruitment and retention and narrowing health equity gaps for Indigenous populations within the region.

Operational approval for this study was endorsed by senior leadership in the Population Health, Human Resources, Clinical Operations, and Medicine and Quality Control departments of IH, and the study received research ethics board approval (H19-02479)

### Indigenous Research Methodology

Indigenous research methodologies (IRM) as decolonization practice (Tuhiwai Smith, 2021) asserts that Indigeneity is integral to informing the research process which evolves from a collective understanding of community aspirations and relational processes (Bourque Bearskin et al., 2016; Kovach, 2009; Kurtz, 2013; Weber-Pillwax, 1999, 2001; Wilson, 2008) and which provide a distinctive frame of reference central to Indigenous worldviews (Weber-Pillwax, 2004; Wilson, 2008). These central tenets as well as overarching values of respect, responsibility, reciprocity, and relevance (Kirkness & Barnhardt, 2001) were used to inform each of the interrelated conversational circles of the research process as described by Kovach (2009) while centering guiding principles of Indigenous Knowledge systems (Weber-Pillwax, 1999).

Grounding ourselves in the protocols of the local First Nations of Secwepemcúl’ecw where this research took place supported our key assumption that distinction-based healthcare is a rights-based approach that leverages the responsibilities, capacities, nature, and lived experience of First Nations, Inuit, and Métis Peoples (Government of Canada, 2021; Tomblin-Murphy et al., 2022). This approach recognizes complex legislative and jurisdictional policies and practices enshrined in health service delivery while honoring Indigenous self-determination and sovereignty in the creation of innovative wellness research (Pidgeon & Riley, 2021) that counters deficit-based research and embeds relational accountabilities to one another.

This research project was designed following key principles of IRM where Indigenous Peoples are leading the research in collaboration with the participants in response to community-based principles, protocols, and practices (Restoule et al., 2018; Tuhiwai Smith, 2021; Weber-Pillwax, 1999; Wilson, 2008). We implemented a distinct relational approach that brought together a local interdisciplinary group composed of highly qualified health professionals and community leaders with expertise in Indigenous languages, research, human resources, Indigenous health, nursing, social work and education. A Sťuxtéws graduate nursing student took a leadership role in gathering Indigenous community members’ support, contributing their unique Indigenous Knowledge, and actively participating in implementing the entire research process, from crafting research questions to managing data, translating findings, and mobilizing research outcomes. In this context, IRM principles articulated by Weber-Pillwax (1999) were applied in the following ways: (a) the interconnectedness of all living and nonliving things was embedded in our collective approach recognizing that the water and ceremonial items carried great spiritual significance; (b) the impact of our motives and intentions on persons and community was central to our relational research purpose of speaking truth to action; (c) the foundation of lived Indigenous experience was embedded into every step of the research process; (d) we grounded the theoretical context in Indigenous epistemology as key relational values and beliefs; (e) the transformative nature of research allowed for deep listening and sharing of vulnerabilities to lessen the burden of feeling isolated and/or harmed by colonial processes; (f) the sacredness and responsibility of maintaining personal and community integrity was to ensure the voices of co-researcher not only captured their employee experiences but suggestions for change; and (g) the use of Secwépemc language and culture as living processes was essential to describing our thoughts and process that aligned with the distinct laws and protocols of the Secwépemc Peoples and was imperative to honoring the local host nation's rights. More broadly these principles guided our ways of working to ensure that our own internal sovereignty, such as self-actualization, self-determination, cultural safety, and wellness-focused knowledge aligned with the values upheld by the research team and the employees of IH involved in this research.

### Methods

Ceremony was our process for creating a space for individuals to engage and share their lived and living experiences of being a First Nations, or Métis health professional. Following Secwépemc research protocols (Gottfriedson,et al., n.d) it was essential for each person to locate, situate and share their intentions: who are you; where do you come from; and why is this work important? As described by Weber-Pillwax, (2021) spiritually informed process that facilitates an internal reconnection of identity and family to conversations on how Indigenous Knowledges and Indigenous health truths have been socially constructed and impacted by our professional practice. In addition, we used critical reflexivity processes to address issues of power and privilege involved in the production of knowledge (Daley, 2010; Morley, 2015). In doing so each of the researchers committed to documenting and expressing their thoughts and vulnerabilities about the impact the research was having on their minds and hearts, in relation to caring for communities (Bourque Bearskin, et al,. 2020). In weekly team meetings, we discussed these politically charged responses so that we were cognizant of how these ideas were shaping our interpretations. Throughout each phase, we built upon these ideas and responses as an iterative process to ensure we were responding to the collective concerns of the co-researchers.

To maintain our relational accountability, we used a talking circle to better understand and facilitate the sharing of stories and experiences (Kurtz, 2013). The talking circles were led by our research team Knowledge Holder Colleen Seymour in partnership with Knowledge Holders Wilfred Grouse Barnes and June Shackley from the local territory where each of the conversational circles took place. It was important for our team to respect local protocol, to share traditional knowledge and teachings in culturally appropriate ways, but also to counter power differential and communication styles to ensure we were supporting co-researchers in culturally safe ways (Lowe et al., 2022).

We recruited a purposive sample of 18 IHP who self-identified as First Nation or Métis and were currently employed or had been employed in IH within the last two years. Each of the three talking circles were recorded and transcribed by an external transcriptionist and then reviewed for accuracy by two research assistants. Due to the sensitivity of data, we only shared anonymized and aggregated data with the research team unless the participant consented specifically to the use of their name in line with principles of data sovereignty as outlined in the consent form.

Using an Indigenous-informed collaborative data analysis process previously implemented by Bartlett et al. (2007), Bourque Bearskin et al. (2016) and Starblanket et al. (2019) which avoids extraction techniques common in western research approaches resonated with our intended relational research design. We honored the stories shared by weaving ceremony throughout the data collection, analysis and mobilization phases. After the independent review of data by two members of the research team, who went through each transcript as a whole document to get a sense of the collective key takeaways, the two researchers then went through line by line to highlight emerging experiences and key takeaway. This was then returned to each circle where the co-researchers completed their own review to ensure accuracy of the data. We achieved consensus in this initial analysis process on what key messages and supporting evidence would be shared to highlight the common experiences expressed by the researchers. We then hosted a final collective circle with all co-researchers and members of the research team to ensure the meanings of stories and experiences of health professionals were accurately interpreted and incorporated into the overall description using their own words. Emerging quotes that informed the collective narrative were further validated, analyzed, and shared with Elders, Knowledge Holders, health care professionals and community stakeholders through local engagement workshops and disseminated to IH and Thompson Rivers University communities of practice.

In a move to honor the co-researchers’ experiences and engagement in this project, their quotes are used extensively in the next section to illustrate the data. By sharing their stories, the co-researchers provided invaluable insights into the emotional and practical realities of being an IHP in a colonial healthcare system. Due to the small sample size and specific locality of the research, co-researchers’ names are not used. These excerpts represent individual contexts that span across time and space and are presented as a piece of the whole story which identifies the challenges of being an Indigenous healthcare professional working towards reconciliation within a colonial health system.

## Findings: In Their Own Words

Speaking from the heart while being in ceremony was transformative in itself. IHP talked about the importance of having sacred space, to sit together and collectively work together to draw on their strengths, rights, relational values, humility and experience for redressing Indigenous-specific racism in BC. The collective story emphasized the profound ancestral responsibilities entailed in their positions that are inseparable from their current day experiences as IHP in mainstream healthcare. Beyond the significant obligations to their patients, they bear an immense responsibility to support their families, communities, and Nations. Their stories offer profound insights into the emotional energy required of these positions. Each individual shared courageous truths that informed the collective story, revealing how colonialism continues to be sustained in their work environments through a deep-seated culture of racism and structural inequity. Three of the five overarching experiences centered on sharing stories about confronting genocide, navigating Indigenous-specific racism, and traversing toxic health environments. The two remaining narratives reaffirmed the co-researchers’ collective message that transformative change must be rooted in the underpinning of Indigenous rights and accountability of employers to uphold these rights.

### Confronting the Perpetual Impact of Genocide

Key thoughts from our conversations highlighted how the legacy of residential schools, intergenerational trauma and Indigenous identity stereotyping perpetually impact IHP in their roles. Co-researchers vividly described their experiences as direct results of the Residential School initiative under the Canadian federal Indian Act (see National Centre for Truth and Reconciliation, 2024). One co-researcher reflected on being a generational bridge, stating:

“I’m the 1st generation that hasn't gone to Residential School. I'm the first person in my family to go to university and my dad went to residential school and I'm the first one who has not.”

Another added their early childhood experience noting: “At five years old I was in the residential school. Temporarily lost the language for a long time.”

A further testimony came from a co-researcher recounting a powerful moment of reclaiming their identity:

My mother went to a residential school over here in Kamloops. And we did the whole reclaiming the Spirit ceremony there last year or two years ago, two years now. We walked across the bridge, up the hill, walked all the way home, we canoed the lake, I rode my horse from IH, it was pretty powerful, yeah. So um, I can go visit over at her office now ……. So now it's just a building cause my aunties and uncles and my mom and all my relatives, their spirits are no longer there. They're home. So, I can walk into that building now and I could still feel the people there that need to be reclaimed but I just pray for them.

All co-researchers shared experiences of intergenerational trauma, exposing the profound and enduring challenges they encounter as IHP. These stories revealed personal hardships and deep-seated motivation that fueled their dedication to their roles as IHP. One First Nations nurse recounted a harrowing incident from her mother's childhood:

A nun tried to kill my mother when she was five years old. She didn’t make her bed properly. The nun took a bedpost and tried to smash my mom's head in with it. My mom had to learn how to walk and talk. They lied to my grandfather and told her that she had polio because she hadn’t come home for one of the holidays. Grandfather went and got her out of the hospital and took her home. She never had to go back. My grandparents never drank until they took my mom. And what my mom had to endure when she went home to her people too because by then the damage is already done in our communities, so she's getting tortured at the residential school and she goes home, and she gets tortured at home now too. You still see this happening in our communities. (First Nations Nurse)

Another co-researcher described the impact of cultural disconnection on parenting:

I wasn’t a well child because my mother didn’t know how to parent. That wasn’t something that was afforded to her. She was removed from her home reserve as a part of a process where the English men were invited to take women off the reserves for companionship until their white wives could show up. Any children you were to have from those unions were to be removed from the reserve to save them from a life of savagery. Unfortunately, my mother doesn’t know which reserve, which community she was taken from. Our life together has been on that quest of her trying to find her connection, her mom and we were a month too late in terms of her ability to do that which led to another journey.

A Métis participant also described the challenges related to legislated identities and stereotyping of mixed ancestry. “You know when you’re raised, well when you’re born of mixed heritage you learn that in some situations, you’re not white enough. And in some situations, you’re not brown enough.” This sentiment captures the complex and challenging reality of navigating identity in a society that imposes rigid categories. These challenges extend to the professional realm when disclosing their Indigeneity to a person of higher authority. This fear stems from potential discrimination and the risk of being misunderstood or marginalized, which can create significant stress and emotional burden. Co-researchers also spoke of the difficulties of exploring and connecting with their own ancestry while working within a colonized health system. The implementation of the First Nations, Inuit, and Métis self-identification program specifically was described as an uncomfortable and awkward experience. One Métis participant shared:

Most people have no idea that I’m Métis until I self-disclose. I have in the past prior to my work in healthcare disclosed and received racism as a result, which baffled me because I didn’t change anything about myself in that other than making a couple of words statement.

The difficulties of self-determination process were further emphasized by a First Nations participant, who pointed out of the inadequacies in how they are asked to identify themselves:

The way they ask you to self-identify is almost, I don’t know, they don’t understand the language, they don’t know how to ask, it's a simple thing of how to ask and to me that almost prevented me from doing my treatment because I just thought oh my God, do I really want to go into this place. *(First Nations)*

These experiences highlight the systemic barriers that IHP encounter as they navigate both their personal identities and professional roles in settings that fail to recognize or accommodate their unique values and cultural perspectives.

### Addressing Indigenous-Specific Racism

IHPs shared many cases of systemic or institutional racism where the dominant colonial perspective clashed or disrespects Indigenous ways of knowing and being. In addition, as a witness and recipient of direct and in-direct racism, IHP expressed a real and greater burden working in a colonial system that remains largely unacknowledged. The emotional burden of being stereotyped as not good enough, and the impact that has on one's identity and belonging was consistently reported. A First Nations co-researcher describes her interaction with other healthcare providers showcasing the fortitude that comes from living through racism.

‘Get me a real nurse’.…And I thought well I was used to being treated like that but I wasn’t happy with it. So, I said OKAY. So, I pushed the buttons on the PA system for the whole building and I announced, ‘Dr. so and so would like a real nurse to emerg stat please and I hung up. This small hospital with only three RN's on, one Emerg, one at each, and so they both came tearing in, they thought something terrible happened because something did. So they said ‘What's going on, what's wrong?’ And I said, ‘Dr. so and so here would like a real nurse. So whoever stays, let me know what needs doing on your wing, so I’ll take over while you’re tending to him.’ And she said, ‘You don’t have to do that.’ And I said, ‘Yes I do.’ I said ‘He's made a choice and so have I.

Other First Nations, and Métis health professionals expressed a distinct feeling of fatigue related to having to continually defend their Indigenous worldview and point out how systemic racism is maintained. This exhaustion is compounded by enduring racist comments and pervasive lack of understanding from non-Indigenous colleagues. As one co-researcher explained:

For me to go out there and try to say anything, educate people constantly, it does get exhausting on top of racial slurs that we get, racial looks and everything in the workplace, everywhere, everywhere, and not only in the workplace, you know, we experience that all over. (First Nations)

Overall, co-researchers reported encountering resistance from supervisors, leadership, and colleagues when they attempted to advocate for change after experiencing or witnessing racism. Several co-researchers highlighted the challenges of speaking up noting there's backlash when you speak out against oppression. Despite these obstacles, the co-researchers shared a resilient outlook:

The days can be tiring, and uh fighting colonialism on a daily basis is hard, it's exhausting. And there's been times when I wanted to quit…but I don't because we all have work to do and we're all here, as it's been said before, because of our ancestors and I have my part to play. (First Nations)

Most of the co-researchers indicated that their needs were not acknowledged, and they did not feel accepted within their work environments, which left them feeling unsupported, excluded, and isolated from coworkers. This isolation was exacerbated by coworkers who often made false assumptions and exhibited bias, leading to further marginalization. One poignant example was shared by a co-researcher who experienced direct racial profiling:

I was racially profiled. I was mistaken for a client. Because of my brown skin I was told I wasn’t allowed to use the staff bathroom. I was accused of being the aggressor in that position…when I took it to my team lead and the manager all they said to me was ‘I’m sorry you experienced it that way. And I said, What way is that? Well I’m sorry you experienced it as racial profiling because she thought she, she thought she was just, you know, doing her job. (First Nations)

Co-researchers articulated that administrators and institutional leaders often failed to effectively address issues of Indigenous identity or provide appropriate guidance on how to respond to the false assumptions. They also reported that co-workers made resentful and hurtful comments in their presence. One First Nation health professional shared her experience of being labeled as a troublemaker for advocating against systemic injustices.

I was labeled the troublemaker and I find that that's really interesting when you’re going up against institutionalized racism, discrimination, and colonization when you do find your voice and you do speak up in a brave way, even though your voice is shaking, you become the labeled one….It takes a lot of courage to break down these racist walls in these institutions. The policies are so racist they don’t even know they’re racist.

This sentiment was echoed by numerous co-researchers who noted that these persistent and insidious comments and inaction had a profound impact on their emotional, mental, and spiritual health, leading to low self-esteem, low job satisfaction, and long-term burnout.

### Uprooting Toxicity and Inequities in Work Settings

Significant contributors to toxic work environments conveyed by the co-researchers were related to organizational values and poor communication. Co-researchers felt unwelcomed, rejected, and minimized in their workplaces. Experiences associated with this theme included bearing the burden which leads to burnout, not having a voice, a lack of empathy, distrust in the healthcare system, and lack of institutional support. They also described a necessary hyper-vigilance as every move and gesture is scrutinized by fellow employees and employers. IHP bear the weight of working in a system that expects them to educate non-Indigenous colleagues about Indigenous ways of being, knowing and doing, yet simultaneously expects them to be silent or compliant, without advocating for being treated with dignity, self-care or spiritual and mental wellness. A participant asked, “how do you encourage people to trust the system that keeps biting you?” Other co-researchers shared similar frustrations:

I just feel like we’re constantly having to advocate for ourselves and speak out but then at the same time certain instances of things come up that happen that almost silence us and makes us not feel safe to speak to certain things that we’re having issues with or conflicts that are coming up. (Métis)

Another First Nation health professional shared*:*

My experiences as a worker in the mainstream health care system has not been positive. I already experienced burnout and almost quit. I was hired as the sole to change an entire system which is very tokenistic and with very little support in place.

Other co-researchers also described being taunted and bullied:

Why can’t you just be satisfied that you have an Aboriginal health program? Why can’t you just be satisfied you have an Aboriginal mental wellness program? Isn’t it enough that you already have an HR plan? It must be fun to eat Bannock and get blanketed and go to pow wows all the time for your work. Patching into calls where I hear leaders of the call talk about being low man on the totem pole. The weirdest aggression for doing a land acknowledgment. (First Nation)

For generations, Indigenous voices have been silenced or minimized as a result of racism, genocide, and systemic oppression. All co-researchers shared feelings of consistently having to self-advocate in a system that inherently does not support a “speak up” culture. There were often feelings of risking employment by speaking up, speaking out, and speaking truth.

During the talking circles, there were sentiments of hope, empowerment, strength, resilience, and optimism shared by co-researchers regarding supportive teams, particularly Indigenous-specific and Indigenous-lead teams, and Indigenous healthcare professionals becoming more vocal. One of the First Nations HP shared her sentiments with a sigh of relief in her closing:

What I see all of you doing is that this is hard work and heart work and it's hard work because of what we face as living Indigenous Peoples with the living experience and it's heart work because we all have a passion to be here because of our families and our communities and our Nations.”

The experiences and stories of our co-researchers signify that toxicity and inequities are embedded in the culture of healthcare. These narratives underscore the urgent need for systemic changes that prioritize Indigenous health education, cultural safety standards and Indigenous-specific racism training within healthcare settings.

### Pathways to Change Through Rights and Responsibilities

Also in the stories two solution-oriented narratives focused on the need to improve access to culture and ceremonies. All participants spoke to the importance of embedding Indigenous healing practices into healthcare, respecting Indigenous ways of knowing and being, and institutions accountability for implementing cultural safety standards. A main feature of the co-researcher stories is the creation of inclusive and supportive healthcare systems, where they feel valued for the knowledge and experience they bring as Pellt'iq't, St’uxwstews, Syilx, Tk'emlúps te Secwe̓pemc, Métis and Cree Peoples reducing barriers for Indigenous healthcare professionals.

#### Upholding Indigenous Human Rights

Indigenous rights about pathways to wellness emerged as a central focus of our collective discussions. Discussions about privileging and normalizing Indigenous forms of healing and knowing surfaced the lack of accessibility to knowledge holders and cultural ceremonies for both patients, families, and employees. Both Métis and First Nations co-researchers emphasized the importance of incorporating both western and Indigenous forms of healing into care planning, the ability to “look after themselves,” and to have support for and access to Indigenous healing practice to nurture their spirit at work at their discretion and without having to seek permission to do so.

I think the odd part is that I have to ask all the time. You know I feel like I need to ask if I can smudge with a client or drum or do a water ceremony, things like these that again seem like things that I should be able to do and shouldn’t have to ask permission to do them. Or feel scared, what if I didn’t ask and I did them, would I get into trouble for doing that?

Asking for permission was a common topic and most co-researchers stated they do it to the best of their ability but are not open or transparent because of the concern over the lack of supporting policy that would support their own practice and what clients are asking for.

I recently was able to do a water ceremony, drum and smudge with a client for our last session which was incredible. I think with a different team lead that probably wouldn’t have happened. So, it looks like a small win I think with that one and again just feeling hopeful that as these conversations happen and we have more advocacy that it will become just a way of being, something that just happens organically and doesn’t require asking permission or feeling like I could get in trouble.

Each person reflected on the significance of their kinship ties and the responsibilities to care and protect each other in a way that causes the least amount of harm, for example:

Being a provider, we always have to go back to our people in our community who are accessing our system of care. We have yet to see the shift on embedding traditional and cultural and social practices across all aspects. So, no matter what door you’re walking through, that should be an option and it should be an option for staff to be supported in our own wellness and again that opportunity to sit in a circle and debrief and we share these stories, I wonder how many of us are out there in the system with no one to talk to.

The focal point of the IHP stories was about accessibility of ways of incorporating traditional health and wellness care into their everyday practice so they are able to care for their patients as well as look after themselves. To have support and access to traditional healing practice is a call that would nurture the personal and professional agency of First Nations, and Métis health professionals. It is within this context that IHP suggest that they are instrumental to implementation of Indigenous health rights, and truth-telling as a collective act of resurgence and resistance to recover from historical acts of Canadian genocide that could have seen the extinction of their families and the destruction of their lands. And to uphold generations of traditional healers, helpers and health professionals who long before us had well designed structures, systems and ways to help people take care of themselves. In deeper reflections, the co-researchers discussed at length the benefits of privileging and normalizing Indigenous ways of healing.

#### Transformational Accountability

What resonated throughout all the conversations with IHP, was challenges faced about commitments to implementing cultural safety standards and to the reconciliation process with local First Nations communities within this specific region. Concerns over the lack of knowledge health leaders had about the impact of colonization and unsafe approaches to working within these populations was even more alarming to some. All co-researchers shared that culturally safe opportunities for debriefing were virtually non-existent, and co-researchers spoke of the lack of leadership and infrastructure to support them in this identified need. Within this context, several ideas emerged, including the need to create safe spaces for people to report incidents of discrimination, educational and professional development, and focused resources for Indigenous Peoples to lead the design and delivery of Indigenous healthcare services. As one co-researcher explained:

We need a cultural safety policy in our organization so that when Indigenous colleagues experience violence at the workplace that they have something to lean on and to hold professions to account. (First Nations)

Many co-researchers also talked about the need to create welcoming places and sacred space both locally and provincially, but everyone mentioned that the level of bureaucracy is often daunting.

…sacred spaces and incorporating traditions into work there's so much red tape that we must go through for something that is just, just should be and should be accepted and supported. I try to have an initiative put forward and it's gone to leadership three times and the final time we had to, there had to be like a briefing note and so it's just the red tape and it's just stuck that it's just so simple and we’re really living up to our commitment as an organization.

Some co-researchers were skeptical:

I don’t think we’re ready for integration because I think we haven’t done the hard stuff yet; we haven’t spoken the truth. And I, when I can’t speak the truth in my own office or like smudge in my own office, there's a problem…nobody wants to talk about the hard stuff…They just don’t and that's where I need to push.

Others reiterated how important it is to note that there were some sites and instances where they have been able to incorporate cultural practices, and where there was support from other First Nations colleagues. For example:

I think of an example of somebody that has come to me for support, and we’ve built a relationship and a friendship, and she works in a facility where she talks about wearing her ribbon skirt every Wednesday. And she talked about it not as though she's being rebellious in doing that but the way that she described to me how she does it and how she incorporates awareness and provides access to cultural practices and things in her work, it's like it's not accepted and she's doing it. I don’t know, not sneakily at all because others know that she does it but it's just not commonplace. And space isn’t being made for that. I think a lot of what needs to change needs to come from the top from leadership. So sacred spaces and incorporating traditions into work, um, shouldn’t require so much red tape and should be something that we can just do.

To bring this section to a close, statements brought up in almost every transcript from the individual stories to the collective conversations was the sentiment of feeling connected and having a sense of belonging and feeling understood:

I am so grateful to be in a circle holding a feather seeing all the medicine here, knowing we came together in a good well. I feel very respected. This doesn’t happen enough, in fact it doesn’t happen at all for me, l unless I do it myself outside of my work setting or hide it in my office.

Another talked about the relational responsibilities and feelings of accountability:

We should be going to the people. We should be getting out of our white towers and getting out there to the community, you know. Um, it's finding a balance between doing that and your job.

Another First Nations HP imparted feelings of belonging:

This is the first time I’ve been in a room with other Indigenous people in the health system talking about things like this in a safe way. A lot of my practice is to help other Indigenous families work through this stuff because I lived it, um, and I have to serve my people and I love what I do.

The IHP desire to support transformative changes in the healthcare system was cited as a collective aspiration but the lack of attention to Indigenous rights and accountability to First Nations, Métis and Inuit populations for unsafe healthcare service in both urban and rural communities was of greatest concern.

Highlighting the unique experiences of being IHP working within the healthcare systems in one health region, was an important opportunity to collaborate as a professional group of healthcare providers. But equally important was to provide insight into the needs of IHP in their efforts to integrate local traditional wellness practices alongside Western biomedical treatments. Through their stories, these IHP shared their truths, not to evoke guilt or shame but to facilitate self-understanding, awareness, and solutions through their own distinct worldviews. Their unique collective experiences serve as a call to action for all health professionals to make more informed decisions to better meet the health needs of First Nations, Métis, and Inuit Peoples. By integrating historical and current colonial challenges, we can facilitate meaningful transformation of Indigenous healthcare service to ensure better outcomes for future generations of IHP and their patients.

## Discussion and Implications

Our research findings from within one health region in BC provide critical insight into the continuing pervasive and enduring impacts of colonization on the wellness of IHP. The stories shared by co-researchers reveal the deep entrenchment of colonialism in the healthcare system despite decades of advocacy for health rights by the Assembly of First Nations, Métis Nations, and Inuit Tapiriit Kanatami organizations (Dyck & Sadik, 2020); numerous litigations in health (Minister of Justice and Attorney General of Canada, 2018); truth-telling initiatives such as the TRC (2015a); and investigations into racism and discrimination in the BC public healthcare system (BC Ministry of Health, 2020). Additionally, the lack of adequate employee supports for retaining a sufficient Indigenous health workforce exacerbates these issues (Lai et al., 2018; Stout, et al. 2021). The IHP stories provide rich insight into working within health structures, systems, and service delivery reinforcing the need for improved Indigenous health research.

### The Persistence of Historical Trauma of Confronting Genocide

A stark example of the ongoing trauma occurred during one of our final research meetings in May 2021, when the Canadian Broadcasting Company (CBC) reported the discovery of 215 unmarked graves at Tk’emlúps te Secwepemc Residential School (2021). This news profoundly affected the entire team, despite its familiarity to most of us. It prompted us to pause our research analysis process to focus on the healing with our families and collectively as a nation. All co-researchers in this study are either first or second-generation residential school survivors or have been directly impacted by the forced separation of families and the erosion of their cultural identities. This event highlighted the lack of structural support within our healthcare ecosystem, as employers struggled to grasp the significance. Consequently, many co-researchers took unpaid personal leave, with some deployed to enhance community support services to address the immense trauma, grief, and loss First Nations, Inuit, and Métis peoples were experiencing. The insufficient attention to the mental health needs of its IHP exemplifies a broader gap in awareness and highlights employment inequities and the taxing emotional burden that some IHP carry.

Despite these ongoing traumas, the event fueled our desire to ensure all members of this research inquiry remain rooted in their homelands, ceremonies, and protocols while caring for their families. Collectively, we viewed it as our responsibility to continue documenting these truths, and more importantly to base our responses on the principles of self-determination and self-governance as distinct rights of First Nations, and Métis populations. This news also served as the impetus for this paper's focus on co-researcher voice to intentionally promote truth-telling before reconciliation and to uphold our relational, reciprocal responsibilities to the members of this research team and in advancing this Indigenous health research. This event has since become a national day of reclamation, now referred to as *Le Estcwicwéy̓—or the missing,* ensuring this history is never forgotten.

Furthermore, our findings add to the body of literature documenting the ongoing Indigenous-specific racism deeply rooted in a history of colonial genocide within the Canadian healthcare system (Boyer, 2017; Browne et al., 2016; FNHA, 2015; Kelm, 1998; McLane et al., 2021; Starblanket, 2018; TRC, 2015b; Woolford, 2015). Addressing and alleviating the social suffering of Indigenous Peoples, both as recipients and providers of healthcare, remains a critical concern for most Indigenous health researchers (Seymour et al., 2023; Simon et al., 2023; Smye et al., 2023). Similarly, all national healthcare organizations are now calling for change, specifically to eliminate Indigenous-specific racism (Canadian Association of Schools of Nursing, 2023; Canadian Institute for Health Information, 2023; Canadian Nurses Association, 2021). This collective response indicates progress and further possibilities to advance research that moves us beyond cultural characteristics and essentialist approaches that are descriptive, and extractive, neglecting the underlying racialization processes that perpetuate systemic racism (Bell, 2023; Hamed et al., 2022).

### Institutional Harms and Systems Transformation

The IHP stories and experiences advance existing research that identifies racism as structurally embedded within healthcare systems. (Browne et al., 2016; Hamed et al., 2022; Horrill et al., 2018; McGibbon & Etowa, 2009). This includes research examining critical race theory, which interrogates the dominance of whiteness and its influence over the biomedicalization of health service delivery (Blanchet Garneau et al., 2021). What we heard from IHP is that despite these insights, there remains limited evidence in healthcare on effective strategies for transforming systems and structures so that policies and programs are embedded in cultural safety standards and are supportive of Indigenous health leadership positions developed in genuine partnership with diverse Indigenous populations, drawing on their lived experiences (Allen et al., 2020; Fridkin et al., 2019). One example of such transformation noted by co-researchers is the restructuring of health service delivery in BC to First Nations communities, as documented by Johnson et al. (2016), which resulted in the formation of Canada's first and only First Nations Health Authority. This complex and committed journey to restoring and revitalizing Indigenous health service through the self-determination process acknowledges the distinct rights held by BC's First Nations communities (FNHA, n.d.). Underpinning this transformation signifies a historic and renewed relationship between First Nations, the government of BC, and the Government of Canada (2023). These partnerships often highlight a range of initiatives and activities from leadership to frontline practice, emphasizing the importance of authentic partnership. Such efforts are part of a long-term transformative learning process, through which First Nations, Inuit, and Métis Peoples are articulating the United Declaration on the Rights of Indigenous Peoples by claiming their health and wellness practices within their knowledge systems, as they have done since time immemorial.

Addressing these issues is crucial as our findings corroborate the evidence that Indigenous health professionals (IHP) endure higher rates of racism and discrimination placing them at significant risk (Durand-Moreau et al., 2022; Phillips-Beck et al., 2020). Our collective takeaway is that solutions must extend beyond cultural safety standards. For IHP to effectively champion Indigenous health as a fundamental human health right it's essential to implement accountability measures that drive systems change, enhance practice standards, refine the policy development process, and promote full, authentic collaboration and partnership agreements.

Moreover, our study highlighted the lack of policy and process at the service level to support all employees encountering instances of harm, racism, and discrimination as reported in the In Plain Site report published by the BC Ministry of Health (2020). Key stakeholders are actively taking accountability and establishing mechanisms to create a safe environment for individuals who experience or report incidents of racism and discrimination ensuring they are adequately supported (Government of Canada, 2024). There is a strong emphasis on fostering inclusivity and drawing from Indigenous health expertise to inform the development of culturally safe practitioners and safe work environments (Greenwood, 2019). Despite these efforts, IHPs continue to encounter significant challenges across the various health settings, from the bedside to the board room, to the classroom, and now extending to the land. IHP often feel marginalized and remain tokenized and othered and continually navigate numerous barriers in their roles as described by Joy-Correll et al. (2022) and Pride et al. (2023).

In response to the growing concerns, BCCNM initiated new measures in 2023 to protect Indigenous patients and Indigenous health employees from retaliation, making a significant step toward addressing harm within the healthcare system. These new standards in cultural safety, humility, and racism are designed to focus on developing critical self-reflexive awareness among health professionals regarding their roles in perpetuating our challenging power dynamic. BCCNM emphasized the importance of truth-telling and holding oneself accountable for redressing issues of power and unrecognized privilege. These practices equip health professionals with the necessary skills to effectively confront the intersection of coloniality and Indigeneity that arise in the workplace. This includes ensuring safety when discussing health professional complicity to ongoing Indigenous-specific racism and responding to such issues in a way that supports a speak-up culture, thereby reinforcing the delivery of safe, ethical, and trauma-informed care that offers immediate relief when immersed in incidents of racism (Graham, 2024).

Our study also sheds light on how systems of oppression operate and perpetuate a culture of silence, detrimentally affecting Indigenous health outcomes. We observe that healthcare service delivery to Indigenous peoples often remains mired in ignorance, blaming, and shaming which contributes to premature mortality (Gunn, n.d.). This is indicative of a broader issue that still occurs in the workplace and speaks to the ongoing evolution of Indigenous health resurgence and resistance to colonial harms (Lavoie et al., 2016). The IHP in this study are calling into question the characteristic approaches of many helping professionals, which are built on Eurocentric ideas of doing the right thing. Yes, these approaches can inadvertently sustain systemic racism. We advocate for critical race theorists to examine the work of whiteness and systemic racism as they are practiced in Canada. This critique aligns with what Gebhard et al. (2022) describe as settler logic, where performative actions and superficial inclusivity are the response to the calls for justice, and this operates under the pretense that tokenistic inclusion of Indigenous Peoples, cultures, and ways of knowing is sufficient to advance reconciliation and anti-racism (Bell, 2023). Drummond's (2023) research extends our critical thinking of professional nurses’ roles and warns of the dangers of performative benevolence which he describes as actions that appear to be doing and saying the right thing but lack any significant and meaningful change and commitment to upholding Indigenous sovereignty. IHP in this study demonstrate this resilience, resistance, and resolve, to un-scripting decades of negative narratives that have rendered Indigenous peoples as risk factors and a burden to society.

As committed partners to change, the IHP in this study expressed the importance of systems research that will advance a better understanding of Indigenous health legislation. As the legislative process unfolds in BC, IHP involvement becomes increasingly important; as the stories demonstrate, IHP have been confronting the colonial health legacy and uprooting toxicity in their work environments perpetuated in everyday acts of unintentional harm that live out in bias-informed care (Wylie & McConkey, 2019). It appears that access and exclusion of Indigenous peoples working within the current healthcare ecosystem remain separated. Jongbloed et al. (2023) explain that:

“natural laws of these territories have been rendered invisible through legislation and socialization. Through the resistance of BC First Nations, we are beginning to see the net of settler-colonialism and our relationship to the net. Keeping our hands, hearts, and minds on this task requires stamina and persistence. It requires everyday discipline for Indigenous-specific anti-racist action. Yet, it is the work of our generation of health leaders” (p. 232).

### Service Change Through Indigenous Rights-Based Healthcare

Our findings also advance the discourse on Indigenous health strengths and rights-based approaches to healthcare service delivery. They extend beyond individual instances of racism to leverage the collective acknowledgment that systemic racism is deeply embedded in healthcare services. This recognition is noted by the Canadian Human Rights Commission (2023) and research by Phillips-Beck et al. (2020) who studied the strengths of community-based primary healthcare service delivery. Our findings relate to the call for leadership to initiate systems support for embracing new models of care inclusive of traditional, spiritual, and territorial education. The multifaceted strategies needed to dismantle these entrenched inequities and culturally unsafe practices discussed by Phillips-Beck et al. (2020) also resonated in the stories of the IHP. They echoed the need for services to be built on the distinct knowledge, values, principles and rights of Indigenous Peoples, positioning Indigenous Peoples with lived experience at the forefront of developing and co-designing Indigenous primary health services (Kennedy et al., 2022).

These changes affirm the sovereign rights of Indigenous communities to redesign their healthcare services which highlights their responsibility to give back to their communities, create accessible healthcare pathways, and serve as role models for future generations. These efforts are crucial to ensure focused improvement on health outcomes, continually asking who is benefiting. Are healthcare outcomes for Indigenous people improving? Is access to health care for Indigenous People becoming more equitable? Are we sustaining a safe and equitable workplace where all health providers actively expose and challenge Indigenous-specific acts of racism?

Finally, to address these concerns and violations of IHP rights to historically and culturally informed working environments, the calls for the re-evaluation of educational strategies, urging an exploration of the pedagogies of genocide and truth-telling (Hantke, 2022). In support, the Canadian Association of Schools of Nursing (2023) has acknowledged the harm in their apology letter, advocating for education and training across healthcare disciplines. This is a fundamental step towards reducing incidents of Indigenous-specific racism in every healthcare organization. These educational initiatives must be collaboratively co-designed as a distinct and unique approach that works alongside Western biomedical practices, ensuring mutual benefits of reciprocal understanding between the different paradigms. This includes questioning whose voices are prioritized in healthcare education and practice, and whether IHPs are adequately supported to fulfill their moral and ethical obligations to uphold Indigenous health sovereignty and lead to the development of culturally appropriate healthcare services. These reflexive questions are essential for transforming the healthcare system into one that genuinely serves and respects the rights and needs of Indigenous people, ensuring their voices are heard and their cultural integrity upheld in every aspect of healthcare delivery.

## Limitations

There are several limitations to this study. There was a small number of co-researchers all based in one health region; thus, the findings may not be generalizable to the wider population of IHP and their work environments. There were only 18 co-researchers from three of the five IH regions. However, the findings are consistent with growing concerns over racism and access to healthcare services expressed in the literature, practice settings and media (Burns-Pieper, 2020; The Brian Sinclair Working Group, 2017; Ward et al., 2021). The research findings do however provide rich contextual information on the unique needs of IHP and offer invaluable perspective where IHP situate their truth as the impetus for action.

Another challenge to completing the research was the impact of the uncovering of unmarked graves at the original Indian residential “school’ located at Tk'emlúps te Secwe̓pemc.” Even though these findings were not new information to most of the co-researchers it had an indirect impact on their practice settings. In addition to the environmental crises of fire that devastated the local region, our ability to host a final in-person circle with all co-researchers was limited. Our final collective analysis meeting shifted to online meetings that were not well attended. The majority of people invited to the final collective analysis responded via email or independently reached out to the lead researcher with their approvals.

## Knowledge Translation Activities

Since the inception of our Indigenous and non-IHP clusters research group in 2018, funded in collaboration with Thompson Rivers University and IH, the team has implemented a number of the collective research goals. As previously described in this paper, one of our main goals was to explore IHP experiences implementing traditional wellness practices into their professional practices. This article represents only one of the four key objectives, the remaining objectives included the following: 1) co-create an Indigenous health professional network; 2) develop a video honoring the voices of IHP and their experiences; 3) develop a partnership with IH to support the co-creation of professional development learning modules for new employees to enhance the new Human Resource strategy plan.

The outcomes of this ongoing research were further supported by the Michael Smith Foundation for Health Research, who provided financial support to complete the remaining knowledge translation activities in 2022. Building on the findings presented in this paper co-researchers continue exploring ways to formalize our IHP working network. In collaboration with the BC Indigenous Health Research Chair program, we began implementing intergenerational mentorship circles and invited Rose Melnyk, a Secwépemc Masters student from the local territory, to become a co-lead of the research team. This knowledge translation project based on their graduate work (Melnyk, 2022) and titled *Indigenous Healthcare Professionals Calls for Change in Interior BC: Applying K̓wseltktnéws and K’nucwentwécws,* expanded our original findings into meaningful local context that resulted in the final 20 calls to change, and is currently in press. Building on this knowledge, we collaborated with a local filmmaker to platform the voices and stories of the co-researchers to inform health organizations about the challenges of being an IHP and of implementing colonial policies that are not responsive to the rights of Indigenous peoples or the cultural needs of distinct communities (in production).

## Conclusion

Over the last five years, the co-researchers continue to learn more about the implementation of distinction-based healthcare in BC. We all have either adopted, implemented, or continue to advocate for transformative change within our respective work environments. One of the key highlights of this research was our collaborative attention to local Indigenous processes of relational responsibility and accountability which guided how we worked with each other in the gathering, synthesis, translation, and mobilization of the collective findings. IHP can provide necessary leadership for creating a larger Indigenous workforce and addressing racism in the healthcare system with their non-Indigenous allies, and Indigenous healthcare professionals are ready to be actively involved. It is crucial to move beyond superficial actions and engage in authentic efforts that acknowledge, respect, and uplift Indigenous voices, Knowledge, and perspectives within the context of health. Health systems must move beyond the colonial view on culture and its connection to health. To redress Indigenous-specific racism in healthcare, the settler logic of white benevolence, whiteness as institutional power, and dominant western health ideologies must be rigorously and authentically examined.

The study identified challenges in the retention of Indigenous health care providers and revealed that culturally safe and relevant health services that value Indigenous knowledge and traditional healing practices was limited. The risk IHP take when working in healthcare systems in fact is of experiencing significant harm, and so the focus needs to shift from recruiting to fixing the systems so employees want to stay working there. All co-researchers reiterated the same message that we must build on what has been started. There is a lot of good work that is happening. As a reminder, a First Nations HP, shared her recent experience as a signal that systems changes are happening:

I'm very grateful and I’m very fortunate to be in a place where our culture and ways of being and practicing is very much respected and utilized on a day-to-day basis. If it's not doing any kind of spiritual care, end-of-life care, smudging, spiritual baths, brushing off ceremonies, water ceremonies they're asking questions, they're asking to participate, and I encourage staff to participate because it's their journey as well. They provide the personal care, they are very involved with the families so when they ask to participate, I very much encourage it. Because it's not only respecting the culture but the people that they're working with. We have lovely nurses and lovely doctors who have their hearts in good places and they’re starting to ask me good questions which gives me hope, it really does. So, I think we’re getting through, you know. (First Nation)

## Notes

1. In the context of this research, self-identified Indigenous healthcare professionals are those who work within healthcare, are distinctly recognized as First Nations, Inuit or Métis, and who traverse various roles including clinical, administrative, leadership, education, and allied positions within settler colonial health systems.

2. At present the term Aboriginal was recently changed to Indigenous as of 2024, but we use in IH Health documents and processes. This is based on the current preference of First Nations and Métis leadership tables as the term is consistent with the Canadian Constitution Section 35(2).
